# Situated Sugarcoating: How Policy Contexts Moderate Teachers’ Strategies for Motivating Student Behavior

**DOI:** 10.1037/e3p0000009

**Published:** 2026-06-04

**Authors:** Madison E. Andrews, Matt S. Giani, Kaitlin Ogden

**Affiliations:** The University of Texas at Austin, 2515 Speedway, Austin, TX 78712

**Keywords:** Situated Expectancy-Value Theory (EVT), College and Career Readiness (CCR), Career and Technical Education (CTE), Industry-Based Certifications (IBCs)

## Abstract

Guided by the lens of the situated expectancy-value theory, we investigate student and educator beliefs about the value of career and technical education (CTE) and industry-based certifications (IBCs). Amid the increasing prioritization of IBCs in state and federal college and career readiness policies, we sought to investigate how students describe their motivation for participating in CTE and IBCs, how educators interpret the value of these experiences and, in turn, shape students’ beliefs, and how the broader college and career readiness sociopolitical context may shape these dynamics. Interviews and focus groups with students (n = 68), educators, and administrators (n = 58) at Texas high schools (k = 7) suggest that students highly emphasize the expectancy, intrinsic, attainment, and utility values of their CTE coursework and view IBCs as a beneficial addition, especially for their perceived utility. Educators echoed students’ sentiments toward CTE but, in contrast, described truly valuable certifications as “the exception” rather than the norm. Despite this skepticism, they felt an obligation to “sugarcoat” the value of IBCs to students in order to meet college and career readiness or accountability goals. Here, we see that IBCs hold different dimensions of value for different stakeholders, such as the distinction between student-level (e.g., IBCs will help them get a better job) versus educator-level (e.g., students’ receipt of IBCs improves school accountability ratings and unlocks incentive funding) utility value. This situated sugarcoating performed by educators may be a key mechanism that links educational policies with student motivation for pursuing tasks educators may not perceive to be valuable for students, like IBCs.

One of the most enduring questions in social science is why people are motivated to pursue particular activities and accomplish certain goals. Expectancy-value theory (EVT) posits that individuals’ expectancies for success, the values they derive from activities, and the costs associated with tasks are the most salient factors shaping human motivation (Eccles, 1983, 1984; Eccles & Wigfield, 2020). An extensive body of literature has been devoted to measuring these components of EVT and demonstrating their ability to predict future outcomes (Eccles & Wigfield, 2020; Eccles & Wigfield, 2023).

Although described as a key component of EVT for decades, less attention has been paid to the broader forces that shape motivation, and in particular how the social, economic, and policy context may moderate meso- and micro-influences on motivation. Critiques of this limitation of EVT have led to a broadening of the framework to emphasize the contextual or *situated* characteristics of motivational forces, referred to as situated expectancy-value theory (SEVT; Wigfield & Eccles, 2020). SEVT outlines how the “cultural milieu” – including gender and other social role systems, stereotypes of activities and the nature of ability, and family demographics – influences socializers’ beliefs and behaviors and constrains the types of activities deemed appropriate or desirable.

While this attention on how the broader social context enables and constrains motivation toward particular activities is warranted, we argue that the indirect relationship between the social context and individual motivation via the perceptions and beliefs of socializers hypothesized in SEVT does not adequately reflect the role of policy in moderating how socializers influence the motivation of the socialized. While SEVT scholars have called for “motivation researchers to engage more with policy makers on the relevance of the work on motivation to policy decisions” (Eccles & Wigfield, 2020, p. 8), we argue that the reverse – the exploration of the relevance of policy decisions to work on motivation – must also be explored. Without explicit attention to the policy context in which socializers shape motivation, neither SEVT nor related motivational theories can fully account for a distinct phenomenon: How socializers motivate others to complete a task, even if the socializers do not perceive value in the activity. In this paper, we elucidate a phenomenon we describe as *situated sugarcoating*. In general, the term sugarcoating refers to the practice of making a task or object superficially attractive or acceptable. We stipulate that *situated sugarcoating* consists of four components: 1) socializers (e.g. teachers, parents, managers) do not perceive an activity to be valuable for the one being socialized (e.g. students, children, employees); 2) socializers are influenced by the broader social, economic, and political context, which does define the activity as valuable to the recipients of socializers’ influence and – more importantly – to socializers themselves; 3) this context leads to socializers motivating others to complete these tasks despite perceiving a lack of value in it; 4) the recipients of socializers’ influence value the activity and are motivated to pursue it.

We examine this phenomenon in the context of an educational policy growing in prominence. Within the college and career readiness (CCR) movement, a growing number of states are encouraging high school students to earn industry-based certifications (IBCs) in high school to demonstrate their readiness for particular careers. IBCs are credentials developed by companies, industry groups, or state certifying entities to verify recipients’ demonstration of particular knowledge and skills. In Texas, where the study is conducted, students’ IBC receipt is factored into several institutional metrics: school accountability rating systems, state-sponsored incentive schemes that financially reward schools for students’ IBC receipt, and professional evaluations and monetary bonuses for career and technical education (CTE) teachers, to name a few. From 2017–2023, the percentage of high school graduates earning IBCs increased from 3–33%. This led to our overarching research question: What motivates students to earn IBCs and educators to promote students’ receipt of IBCs?

Through interviews and focus groups with students (*n=68*), educators and administrators *(n=58)* at seven Texas high schools, we find that educators perceive limited intrinsic, attainment, and utility value for students for the majority of IBCs that count in state policy. However, educators readily admit that the policy context exerts a powerful influence over their beliefs and behaviors in ways that incline them to encourage students to earn IBCs. In short, we find considerable evidence of “situated sugarcoating” – educators over-emphasizing the benefits and understating the costs of IBCs in order to motivate students, which is strongly shaped by the particular social, economic, and policy environment.

Our paper is outlined as follows. We first review literature on CTE and IBCs, both in terms of the policies that shape these educational activities as well as research on why students are motivated to participate in CTE and earn IBCs. We then explore the SEVT framework we use to guide our research, and provide more detail on our methods before presenting our results. We conclude by discussing the implications of our findings for CTE and IBC policy specifically, as well as the significance of *situated sugarcoating* as a mechanism that may explain motivation toward sub-optimal outcomes.

## 1. Literature Review

### 1.1. College and Career Readiness Policy Context

In recent years, policy interest and research related to CCR for high school students has surged (Darling-Hammond et al., 2014), in part due to graduates facing challenges successfully navigating the labor market (Carnevale et al., 2013), critiques of the “college for all” paradigm (Rosenbaum, 2001; Rosenbaum et al., 2015), and growing public skepticism of the value of higher education amid rising costs (Pew Research Center, 2024). For over a half-century, CTE, historically referred to as vocational education, has been one of the most prominent strategies for preparing students for the labor market (Dougherty & Lombardi, 2016), with more than 82% of schools offering CTE courses and 88% of students earning at least one CTE credit (National Center for Education Statistics at IES, 2020). In the past decade, CTE has been linked to a new CCR strategy rising to prominence: the receipt of IBCs.

The increasing prevalence of IBCs in high schools is a result of federal and state policy reforms that incorporate components of CCR into state accountability plans and funding systems, with some policies offering financial incentives for schools based on students’ receipt of IBCs (Advance CTE & The College in High School Alliance, 2022; National Center for Education Statistics at IES, 2020). The Every Student Succeeds Act (ESSA, 2015) placed new requirements on states to incorporate components of college and career readiness, like CTE and IBCs, into state accountability plans (Hackmann et al., 2019), and by 2023, at least 16 states had begun using IBC receipt as an indicator of CCR in their ESSA plans (Education Commission of the States, 2023). Perkins legislation (Perkins V, 2018) made the attainment of recognized post-secondary credentials, including IBCs, one of the core accountability indicators of student performance in secondary CTE, and at least 22 states chose this indicator.

The Texas school accountability system relies on measures referred to as College, Career, and Military Readiness (CCMR) indicators. CCMR indicators include being college-ready (e.g., earning an Associate’s degree or completing a dual-credit course), career-ready (e.g., earning an IBC or completing a CTE course sequence), or military-ready (e.g., enlisting in the United States Armed Forces). IBCs were added to these indicators via House Bill 22 (2017), when the Texas Legislature directed the Texas Education Agency (TEA) to factor students’ receipt of approved IBCs into the state’s public school accountability system and to publish a list of approved IBCs (Texas Education Agency, 2024b). Since 2019, Texas schools also receive monetary “CCMR outcomes bonuses” for students meeting specific CCMR indicators, with $186,000,000 and $197,000,000 distributed across the state in 2021 and 2022, respectively (Texas Education Agency, 2024a). Students who earn IBCs qualify for CCMR bonus funds under the career-ready criteria, whereas students must enroll in a postsecondary institution for schools to receive bonus funding under the college-ready criteria. By design, these federal policy reforms and state accountability systems have the potential to over-incentivize IBC receipt, regardless of IBCs’ personal value to students.

### 1.2. Career and Technical Education

CTE courses are designed to “focus on the skills and knowledge required for specific jobs or fields of work” (National Center for Education Statistics at IES, 2024) in order to “provide both a bridge to well-paying jobs for non-college bound students, and a head start on more advanced career training for those who seek a college degree” (Carruthers et al., 2024, p. 2). And indeed, research shows that CTE participation has been linked to positive education and workforce outcomes, including an increased likelihood of high school attendance (Plasman et al., 2024), high school graduation (Brunner et al., 2023; Dougherty, 2018; Ferreira & Martins, 2023; Gottfried & Plasman, 2018; Lindsay et al., 2024), college enrollment (Brunner et al., 2023), short-term employment (Lindsay et al., 2024), and short-term wage returns (Brunner et al., 2023; Hendricks et al., 2021; Kreisman & Stange, 2020).

While research suggests that CTE participation improves students’ postsecondary outcomes, concern about CTE’s impact on employment outcomes lends support for the growing emphasis on IBCs. Despite CTE’s central role of preparing students for the labor market, there remains a lack of rigorous evidence suggesting that CTE participation improves long-term earnings (Lindsay et al., 2024). Further, initial labor market benefits may fade over time, particularly if CTE students are unable to enter careers with opportunities for advancement or to “upskill” over time (Hanushek et al., 2017). Thus, ensuring CTE programs provide students with verifiable skills or certifications sought by employers remains a key priority, and IBCs are posed as one potential solution to these concerns.

### 1.3. Industry-Based Certifications

Increasingly, CTE courses include opportunities for students to earn IBCs (Giani, 2022; Xu & Backes, 2023), with more than half of states having implemented policies that allow students to earn IBCs in CTE (Advance CTE & The Association for Career and Technical Education, 2024). The logic of IBCs is that coursework alone may be a weak signal to employers that prospective employees possess the knowledge and skills needed to perform particular job functions. Like participating in CTE, earning IBCs in high school has been linked to positive education and workforce outcomes, including an increased likelihood of high school graduation (Glennie et al., 2017; Walsh et al., 2019), college enrollment (Glennie et al., 2023; Glennie et al., 2020; Walsh et al., 2019), college graduation (Glennie et al., 2023; Glennie et al., 2020), short-term employment (Baird et al., 2022; Giani, 2022; Turley, 2023; Xu et al., 2024), and short-term wage returns (Baird et al., 2022; Giani, 2022; Hendricks et al., 2021; Walsh et al., 2019; Xu et al., 2024). Further, research suggests students’ completion of related CTE courses and IBCs is positively correlated with affective benefits, like students’ sense of self-efficacy in their ability to persevere in overcoming challenges and their performance on various tasks (Spillers & Lovett, 2022).

However, when analyses that explore the benefits of earning an IBC in high school are broken down by outcome, subject area, and student characteristics, the value of IBCs appears less definitive. For instance, the relationships between IBC receipt and postsecondary and workforce outcomes vary across IBC subject area (Giani, 2022; Xu et al., 2024). There is evidence of gender segregation in IBC participation that aligns to gendered workforce patterns and evidence that students’ academic achievement levels influence which IBC exams they take (Eagan & Koedel, 2021). Further, research suggests not only that there is a limited relationship between IBC receipt and labor-market needs (Dalton et al., 2021), meaning students may not be earning credentials that actually provide entry points into in-demand fields, but also that the majority of IBC earners were neither enrolled in a major nor employed in an industry after high school that matched their IBCs (Giani, 2022), further limiting the potential long-term value of these credentials.

### 1.4. Students’ and Educators’ Perceptions of CTE & IBCs

One critical gap in our understanding of the benefits and value of both CTE and IBCs is the lack of qualitative research on students’ and educators’ perceptions of CTE and IBCs. Most of the extant literature consists of quasi-experimental, large-scale quantitative studies that focus on the educational and labor market outcomes related to CTE and IBCs. While these studies provide valuable insights, they do not capture students’ motivation for participation in CTE and IBCs, educators’ roles in shaping these CCR experiences, or how schools’ responses to policy mandates may mediate these dynamics – we know that students are increasingly participating in these opportunities in high school, but we do not know what motivates students or what they view as the costs and benefits of participation.

Several researchers have called for more qualitative research to understand the reasons students pursue CTE and IBCs, as well as external “push” and “pull” elements outside of students’ educational or career goals that may influence student participation (Ansel et al., 2022; Carruthers et al., 2024; Dalton et al., 2021; Glennie et al., 2023; Jacob & Ricks, 2023; Xu et al., 2024). Students could “be “pushed” into obtaining a certification if institutional pressure or policy levers strongly encourage students to earn certifications (Dalton et al., 2021, p. 6).

For example, the viewpoints of CTE teachers, school counselors, and administrators may play a crucial role in shaping students’ understanding of and subsequent interest in pursuing CTE and IBCs. Previous research on educational stakeholders’ beliefs about CTE indicates that educators’ perspectives on its purpose and value can sometimes lead to misrepresentations in comparison to other college and career readiness pathways (Cashdollar, 2023); in their eagerness to highlight the advantages of CTE courses, such as “low-risk, high-reward learning opportunities,” educators downplayed the relative advantages of other careers that required postsecondary degrees (Cashdollar, 2023, p. 1716). These biases may in turn influence students’ abilities to make informed and accurate decisions about their career aspirations. However, this phenomenon is not well documented, and students’ voices have remained largely absent from these examinations. Our investigation was guided by three questions:

1. How do students perceive the value of CTE courses and IBCs, respectively?
2. How do educators perceive the value of CTE courses and IBCs, respectively?
  - Do educators perceive different dimensions of value for students, for themselves, and for their schools?
3. How do educators motivate students to earn IBCs, and how is this influenced by the policy context?

## 2. Theoretical Framing: Situated Expectancy-Value Theory

We investigate student and educator perceptions of CTE and IBCs using the lens of situated expectancy-value theory (SEVT), an updated version of the original expectancy-value theory (EVT), which posits that students’ motivations and behaviors are influenced by the expectations and values they associate with completing tasks along their educational pathways (Eccles, 1983, 1984; Eccles & Wigfield, 2020). Originally specific to adolescents in mathematics, EVT was developed to understand students’ achievement related choices, and has since been applied in the study of career choice and persistence across various educational contexts (Eccles & Wigfield, 2020; Eccles & Wigfield, 2023; Wigfield & Eccles, 2020).

EVT posits that “individuals’ expectancies for success (ESs) and subjective task values (STVs) are the most proximal psychological determinants of task and activity choice, performance, and engagement in the chosen activities” (Eccles, 1983; Eccles & Wigfield, 2020, p. 3). Expectancies for success, which is very similar to Bandura’s (1997) self-efficacy construct, can be defined as an individual’s belief in their ability to be successful at a task. Subjective task values are delineated into intrinsic value, utility value, attainment value, and cost. Intrinsic value, or the expected enjoyment of engaging in a task is commonly referred to as interest. Utility value is how useful a task is to an individual’s current or long-term goals. Attainment value is a measure of the perceived personal importance of a task, or how important completing a task successfully is to an individual. Finally, cost refers to the possible sacrifices required to complete the task, and is sometimes disaggregated into effort cost, opportunity costs, and emotional costs.

In the last decade, Eccles & Wigfield have articulated their support for moving towards a situated expectancy-value theory (SEVT), which recognizes that ESs and STVs are situated with time and place (Eccles & Wigfield, 2020). In other words, SEVT also acknowledges the “social, contextual, and psychological factors that influence which specific aspects of each STV components are weighted most heavily as individuals assess the relative STV of various short- and long-term activity options” in any specific context (Eccles & Wigfield, 2020, p. 6); these factors could include things like students’ personal identities and identity beliefs, prior experiences, peer and parental influences, school and classroom characteristics, and broader cultural contexts, to name a few. This reframing as SEVT also emphasizes that individuals will assign and develop their own hierarchies of, or relative importance of, ESs and STVs of certain tasks in ways that are “both situationally specific and culturally bound” (Eccles & Wigfield, 2020, p. 2).

Although this sharpening focus on how the social and cultural context shapes motivational influences is a positive development, SEVT scholars rarely engage with how the policy contexts shapes motivation, particularly in regards to moderating the relationship between socializers and those whom they are trying to motivate. In particular, SEVT does not elucidate potential mechanisms that may explain situations in which socializers motivate others to complete a task, even if they do not perceive that task to be valuable. This study is designed to examine these mechanisms by exploring students’ and educators’ ESs and STVs relative to CTE courses and IBC exams within the broader sociopolitical context of CCR policy reforms.

### 2.1. Applying SEVT to CTE Pathways and IBCs

Thus far, SEVT’s application in CTE and IBC research has been limited. One study used EVT to analyze students’ motivations for participating in CTE programs in a sample of rural schools, finding that students prioritized courses that offered paths to shorter-term financial benefits via accelerated entry into the workforce (Newman, 2024). Another examined students’ likelihood of concentrating in computing CTE pathways specifically, and found no association between math/science expectancy-value constructs and students’ computing course-taking, indicating that EVT did not adequately capture the factors that motivate students to take computing courses (Perry & Montoya, 2024); however, this study relied on survey data rather than interview data. Hayes (2021) explored community college students’ perceptions of the value of their CTE experiences, revealing that students recognized intrinsic value through the practical, hands-on nature of the classroom experiences and identified attainment and utility value in the importance they placed on completing CTE programs to achieve their future career goals (Hayes, 2021). To our knowledge, no studies have incorporated the “situated” component of SEVT to examine CTE or IBCs, suggesting that additional research is necessary to better refine how SEVT can be applied to understand students’ motivations in CTE programs, the focus of the present paper.

We anticipate that students will build ESs and recognize all four STVs via their engagement in CTE and completion of IBCs; we hypothesize that these curricular experiences will increase students’ confidence in their abilities to successfully complete tasks (i.e., expectancies for success), especially when their skill-building experiences align to students’ career interests (i.e., intrinsic value). We expect students to place a high level of importance on mastering CTE content and passing IBC exams (i.e., attainment value), due to their ability to facilitate immediate employment or postsecondary goals (i.e. utility value). Because CTE and IBC participation in Texas is largely financed by schools or districts, rather than by students directly, we expect students to identify minimal costs associated with these experiences.

To better understand the context in which students develop ESs and STVs relative to CTE and IBCs, we also investigate educators’ perceptions. While we expect students and educators to align on some beliefs, we anticipate that educators’ perspectives will be more saliently influenced by the broader school, district, and state-level factors that shape CTE and IBC offerings, and thus that their perceptions of the value of these offerings may manifest along different dimensions than students identify. For instance, the utility value of IBCs for students likely relates to how IBCs help them accomplish their career goals, but the utility value of IBC receipt for educators could relate to how IBCs help schools and educators accomplish accountability and funding goals, perhaps regardless of the utility value of IBCs for students. If this is the case, we further anticipate that as educators communicate the value of these offerings to students, the personal and school-level values that educators recognize in IBCs may supersede their beliefs about the student-level value, and lead to misrepresented or overinflated promotion of IBCs. In other words, we broadly hypothesize that the CCR policy, funding, and accountability landscape may over-incentivize IBC participation via situated sugarcoating.

## 3. Methods

### 3.1. Overview

The rationale for this study builds upon our prior work (Andrews et al., 2026; Giani et al., 2025) with quantitative data from Texas’s statewide longitudinal data system, the Texas Education Research Center (ERC). The ERC includes individual-level data on every K-12 public school student from the Texas Education Agency (TEA), including records of students’ high school course-taking, IBCs earned, student characteristics, school information, and district information. Descriptive analyses of a sample of more than two million students (*n=*2,119,750) who graduated from high schools (*k=*1,982) in Texas between 2017–2022 revealed a significant increase in the completion of IBCs in high school, rising from 3% to 33% of students earning a certification from 2017 to 2023. Simultaneously, the rate of IBC alignment to students’ CTE coursework (i.e., whether students earn IBCs in the same subject area as their CTE studies) dropped from 43% to 27%. Considering these findings in the context of growing emphasis on IBCs in state and federal policy, we sought to investigate how students describe their motivation for pursuing CTE and IBCs, how educators shape students’ beliefs about CTE and IBCs, and how the broader CCR sociopolitical context might moderate these processes.

To address our qualitative research questions, we conducted site visits at seven high schools. At each school, we engaged with a range of CTE students, CTE teachers, and other school staff, like counselors and administrators. Conversations with students investigated their experiences in and perceptions of the value of CTE courses and IBCs. Conversations with educators investigated their opinions of and experiences with CTE courses, incorporating IBCs into CTE courses, and the value of CTE and IBCs for students.

### 3.2. Data Collection

All public high schools in Texas were eligible to participate in this study. However, this work is part of an ongoing study where we categorized high schools into a policy implementation typology by students’ rates of CTE and IBC completion (Giani et al., 2025). We categorized each school as having High- (>50%), Low- (1–49%), or No-Certification rates and High- (>50%), Low- (1–49%), or No-Alignment, resulting in a 6-category typology that we descriptively titled: No Certifications (Uninvolved), No Alignment (Unaligned), Low Certifications-Low Alignment (Sporadic), Low Certifications-High Alignment (Limited, But Strategic), High Certifications-Low Alignment (Gaming the System), and High Certifications-High Alignment (High Performing). The high schools included in this qualitative investigation were strategically recruited across these categories. Recruitment contacts were principals, vice principals, counselors, and CTE educators identified from the public websites of each school. Recruitment materials, sent via email, advertised opportunities for students and educators to participate in paid, hour-long interviews and focus groups.

We conducted interviews and focus groups with students (*n=68*), educators, and administrators *(n=58)* at *(k=7)* Texas high schools to understand student and educator perceptions of CTE and IBC experiences. The choice between interviews and focus groups depended on participant availability and campus scheduling. Because the same questions and topics guided both formats, the data were comparable and could be analyzed together to identify shared themes across participants.

All student participants took part in focus groups; we conducted a total of twelve student focus groups, with an average size of 5.6 participants. When possible, students were grouped with other students in their CTE program, but groups were sometimes combined to accommodate students’ schedules. Most CTE teachers participated in focus groups; we conducted 9 teacher focus groups, with an average size of 4.4 participants. Individual interviews were conducted with educators if they were unavailable for the focus group timeslot; 4 individual interviews were conducted. All administrators and counselors took part in one of six focus groups, with an average size of 2.3 participants. We separated these focus groups from the CTE teachers to ensure teachers were not limited in their responses by the presence of their administrators.

Informed consent was obtained from all participants at the beginning of each focus group or interview; in cases where student participants were under 18 years of age, parental/guardian informed consent was obtained in advance of the interview. This study was conducted in compliance with the Institutional Review Board at a large research institution in the Southwest. Tables A1–3 in the appendix detail our semi-structured interview and focus group protocols and their alignment to our theoretical framework, SEVT. All conversations were recorded and then transcribed for analysis.

Table 1 provides an overview of our participants and their schools’ characteristics, including campus size, region, and the percentages of all students at each school that earn IBCs, participate in CTE, and concentrate in CTE pathways (i.e. take three or more courses in the same subject).

### 3.3. Data Analysis

Qualitative data analysis began at the beginning of the study and continued throughout, using deductive, inductive, and thematic analytical techniques (Braun & Clarke, 2022). This was intended as a process of “making sense of the data.. . [which] involves consolidating, reducing, and interpreting what people have said and what the researcher has seen and read—it is the process of making meaning” (Merriam, 1998, p. 178). Immediately after conducting each interview, the researchers wrote an analytical memo about the conversation. These memos serve as a “code- and category-generating method” that allowed us to obtain a broad sense of participants ‘experiences and to begin to draw comparisons across groups (Saldana, 2009, p. 50). We continued our analysis by reading cleaned transcripts and adding comments to capture our emergent reflections (Creswell & Poth, 2018). This process allowed us to identify the basic concepts present in the dataset, building a vocabulary of our data (Saldana, 2009). We then used focused coding to categorize the data by applying a priori (theoretic) codes from SEVT (Miles et al., 2020); “focused coding searches for the most frequent and significant initial codes to develop the most salient categories in the data corpus” (Saldana, 2009, p. 156).

### 3.4. Transparency and Openness

Materials and analysis code for this study are not available. The quantitative data underlying this article were provided by the Texas Education Research Center’s (ERC) statewide longitudinal data system. The quantitative and qualitative data cannot be shared publicly for the privacy of individuals in each dataset. The code used to create the analytic data files in the ERC and produce the typology are available upon request to the corresponding author.

## 4. Findings

### 4.1. Overview

Summarized in Table 2, our analyses reveal that both students and educators recognize dimensions of value in CTE coursework and industry-based certifications, but their perceptions of those values differ. Students heavily emphasize the expectancy, intrinsic, attainment and utility values of their CTE coursework, and frame IBCs as a beneficial add-on to those classroom experiences, especially in terms of their attainment value and utility value towards their career goals. Students note costs affiliated with completing both CTE and IBCs, but these tradeoffs were framed as minimal in comparison to the benefits, and for IBCs specifically, the costs were not salient for most students.

Educators note the expectancy, intrinsic, attainment and utility value of CTE experiences *for students (i.e. student-level)*, but do not generally apply those value beliefs to IBCs. Educators feel that the inclusion of and emphasis on IBC exams actually dampens the intrinsic and utility value of CTE experiences for students, as class time is diverted towards test preparation. There is a general consensus among educators that few certifications are well aligned to industry needs, and therefore IBCs do not actually provide students with the skillsets or skill-signaling required for educators to see their utility value for students.

However, educators also see costs, attainment and utility value in IBCs *for themselves and their schools (i.e. educator-level)*, where the certifications are metrics for incentive allotments or indicators of teacher performance, or are a means for accomplishing school-level accountability goals. At times, this leads to educators overemphasizing or misrepresenting the value of IBCs for students to their students. Next, we provide an overview of student and educator beliefs about CTE and IBCs relative to each of the tenets of SEVT. We begin with students’ views, and then educators’, which are delineated into student-level values and educator-level values.

### 4.2. Students’ Perspectives

#### 4.2.1. Expectancies for Success

Many students spoke about how CTE courses provided scaffolding for them to build confidence in their skills and in themselves. Students felt that the CTE classes “taught personal life skills” and “career-based skills”—skills like communication, time-management, goal setting, and customer service which made them more confident in their abilities to succeed after high school. CTE classes tended to be smaller than other courses, and students reflected that these classes were “more private […] it was easier to learn because there wasn’t [*sic*] so many people in a classroom.” Students especially valued that CTE was “a safe space to make mistakes.” The smaller class sizes also allowed students to develop personal relationships with their teachers and other professional mentors who visited their classes, with several students remarking on how CTE teachers did a great job of “not only understanding us as a student but as a person.”

The CTE courses students selected typically aligned with their post-graduation career interests, and so completing hands-on coursework gave students a sense of “how it was going to be in the real world” and confidence in their abilities to both perform tasks and get a job in their chosen fields. One law enforcement student stated they “would confidently say, if I would have to go be a cop right now, I could be confident in riding along with someone and knowing what I’m doing in situations.” In all, students’ CTE experiences contributed to confidence in their soft and professional skillsets and their ability to succeed in their chosen career paths. Similarly, IBC exams seemed to offer a small boost in terms of expectancy, with students noting their confidence grew from feeling they had “a leg up in the real world” and that the certifications “would look really good on applications.” Students’ experiences preparing for and passing IBCs gave them a sense that “you know what you’re doing, you got an idea. And that can put you ahead of somebody else who’s going for the same job and might not have those certifications.”

#### 4.2.2. Intrinsic Value

Students and educators saw the intrinsic value of CTE coursework and experiences as inherent to the programs themselves, as most students were selecting CTE courses based on their interest in the subject matter, either as a long-term career goal or a form of career exploration. Students reflected on how much fun they had being creative in CTE courses, saying “it doesn’t feel like a class. Class is boring. I would compare it to recess if I was a little kid. I don’t feel like it’s a class.” Students find these courses to be “very valuable” and “more engaging” than core coursework due to the “hands-on” experiences and “interactive projects.” However, the addition of IBCs sometimes added “test prep” and “book work” that students did not find as interesting.

In comparing CTE to core coursework, one student in a Health Science pathway reflected that sharing career interests with her classmates had substantial benefits for her socially and academically, where she and her classmates:

…really connected on our love for science and for our love of healthcare and wanting to go into this field. It was really nice to find a group of people that could really share that deep interest and career path that you wanted to also pursue, instead of just having a bond over a book in a class. So, you shared this deeper level connection with these people that then followed you throughout all of these courses. You could create study groups and apply to colleges together.

#### 4.2.3. Attainment Value

For students, attainment value, or the importance of completing CTE courses and IBCs successfully, primarily appeared in conversations about monetizing the skillsets gained through CTE experiences and IBC licenses. Thus, the attainment value of CTE and IBCs largely emerged as a secondary effect of utility value. One student spoke about how their CTE coursework contained “opportunities for pretty much anybody. I was building a nightstand last year and my teacher was encouraging me to go to competitions and sell them and make money, like scholarships. And so, I think [we’re] motivated by that chance to make money off of whatever [we’re] doing, especially when [we’re] doing what [we] love.”

In comparing courses with and without IBCs, students indicated that classes where you earn a certification were more important to them than “other classes [where] you just take it to take it or just to say you took it.” The exams did seem to add pressure onto students, however, especially when successfully passing the exam was a requirement for their career goals, such as earning a Cosmetology Esthetician License so one student could “open [her] own shop after high school.” One student reflected on having “a lot of nerves” regarding the test he and his peers would be taking the next week, but reassured his classmates in saying: “If it’s something we really want to do and we truly have a passion for it, then you know what I mean? We should feel brave and we should feel good about taking the test.” These pre-exam anxieties could indicate that success on the certification exam was critically important to students.

#### 4.2.4. Utility Value

The utility value of both CTE experiences and IBCs was a prominent thread in our conversations with students. In addition to the aforementioned confidence boosts students noted from building skills like communication, customer service, goal-setting, self-motivation, and time-management, students also indicated that they felt these skills were personally and professionally valuable in helping them achieve their current and long-term goals. Similarly, students noted that taking CTE courses that aligned to their various career interests was not only more enjoyable, but also provided utility value in exploring, concentrating in, and becoming more aware of career options in a setting where the costs of career exploration and concentration were minimized. One student recommended CTE for all students because the courses “really help you open your eyes on opportunities and things that you can get into in the future.”

Students who felt certain of their career goals utilized CTE course pathways to concentrate in their field of interest, with one student remarking that “I know I want to go to college about [Agriculture]. I’m not sure which major yet, but I know it’s about Ag and I took a lot of Ag classes, so I think it’s going to help me and give me a head start.” For these students, CTE courses had the added benefit of being “a class that I know that I’m going to use after this that will lead me to where I want to be. And so, it’s a little more rewarding.” Students felt these benefits were amplified by IBCs, noting that the combination of CTE and IBCs

“definitely gives us an advantage too, compared to other people. If you didn’t have these classes in high school and you had to start out, you’d have to pay for it yourself. But because we’re in CTE classes, the school pays for all of the certificates and stuff and testing that we have to get. And so, it makes it easier for us to be already ahead in the career that we want to do.”

Most students valued that IBCs could give them a “leg up in the real world” and “more chances of getting hired because [they] have more experience and [IBCs] under [their] belt.” Many intended to pursue additional education after graduation but felt that IBCs gave them “really good plan Bs… so if anything doesn’t work out with higher education, we have something to fall back on. We don’t have nothing coming out of high school.” However, students seemed to value the skills over the IBC itself, saying that “even if you took it and you didn’t pass, it was still very beneficial, because you gained knowledge and skills just from preparing for that test.” Several students agreed that they would have completed the CTE program without an offered IBC, because the experience of the course was so valuable to them.

#### 4.2.5. Costs

Students reflected on the opportunity and financial costs associated with pursuing CTE pathways and preparing for IBC exams, including making curricular tradeoffs between core, dual-credit, AP/IB, and CTE course offerings. In cases where schools required students to concentrate in CTE to graduate, some students expressed frustration with limited pathway flexibility, where “you can’t really switch into something else” after declaring a concentration. Completing CTE coursework and preparing for IBC exams requires students to balance their academic, social, and familial obligations, but students seemed to believe that the work was well worth it.

In most cases, the financial costs of the certification exams were covered by the school or district, although some students were faced with the potential of out-of-pocket costs. For instance, one administrator explained that students in their Health Sciences CTE program received classroom training for both the Certified Medical Assistant (CMA) exam and a Phlebotomy Certification, but only the CMA exam cost would be covered; “if they would like to get that extra certification, our teachers work with them on that, but it’s not something that’s necessarily covered by the district.”

Other students were harmed by policy changes that shifted which certification expenses could be covered, leaving students uncertified and extremely frustrated. One Education student had completed “three classes – two prerequisites, then a class” to qualify and train for a Child Development Associate (CDA) exam, only to find that the district would no longer pay for the exam by the time she had completed the third course. She reflected that “I would’ve taken the CDA if the school paid for it. They said they would and then they went back on it. And it’s $300. So, I can’t just go and take it.” She remarked that the policy change “discounted all of the work that my friend and I had done” and that “if we had known, we would’ve picked a different pathway.”

### 4.3. Educators’ Perspectives

Educators described both the dimensions of value that CTE and IBCs had to students, and to educators or schools. For example, there is a distinction between student-level utility value (e.g., IBCs will help them get a better job) and educator-level utility value (e.g., students’ receipt of IBCs improves school accountability ratings and unlocks incentive funding). We delineate these concepts via headers below, as seen in Table 2. Educators did not express multiple levels of value for all concepts (e.g. expectancies for success). For these factors, we describe only the student-level themes.

#### 4.3.1. Student-Level Expectancies for Success

Educators echoed students’ sentiments that CTE courses offer a more supportive environment for students, remarking specifically on how CTE courses “encouraged [students] to make their own community.” In these environments, they describe seeing students thrive while developing soft and technical skills. They reflected on the benefits of having CTE programs at high schools, because these courses “validate students. Every student doesn’t want to go to college, but their pathway and what they want to do is equally as important. I think programs like ours validate what they really want to do, what their pathway and purpose is.” In reflecting on the addition of IBCs into CTE coursework, teachers felt that “some of the kids get a little bit of a boost out of it, an ego boost. That’s a good thing, I think.”

#### 4.3.2. Student-Level Intrinsic Value

CTE teachers see similar intrinsic value in the coursework they teach, noting again that CTE courses tend to be more hands-on and interactive. They know that students find CTE courses more interesting than non-elective courses and intentionally design learning experiences around the belief that “it has to be fun. If we’re being honest, we’re the reason they come to school. It ain’t because of their English and Math class.” Educators value the flexibility of CTE courses to allow space for “the creativity, the ability to be relaxed and let them actually apply [their skills],” but with the addition of IBC exams, they wrestle with balancing teaching CTE curriculum and teaching to the IBC exam, equating the certifications to mandatory end-of-course exams used in school accountability. They feel that “the application is missing because I’m trying to teach to this test.” In other words, CTE teachers see the intrinsic value of CTE experiences, namely the hands-on, creative application of course content, being dampened by the addition of IBCs.

#### 4.3.3. Student-Level Attainment Value

Because CTE courses incorporate project-based learning, there were many examples of educators describing students’ completion of these projects as valuable, and CTE teachers saw the importance of students’ successfully mastering these skills as both inherent to their programs and paramount to students’ future success. Some teachers described themselves as “realists, and that’s what we try and give the students - real world experiences.” Educators echoed students’ sentiments about the importance of doing well on IBC exams in order to gain access to career opportunities, saying that they tried to advertise the importance of passing IBC exams, because the certifications can provide students with access to “a position where you can be successful and provide for yourself, or it’s a great steppingstone to move forward in [their field].”

They also felt it was important to give students recognition for passing IBCs, with one school implementing a “certification ceremony” where students and their families gather to “hear their name called, and the parents get to hear the value of what their kid has attained.” Some educators even expressed a belief that the value of students attaining the certification itself was more important than the potential utility value of the IBC, saying:

We’ve seen kids in classrooms that are on the college route, they know for sure, “Hey, I’m going here, this is what my goal is.” But then we also have the kid that has no idea what they want to do, and so […] being able to give them something to walk away with has shifted my perspective of if there’s a “need” in the industry [for that IBC]. There’s a need for these kids to understand the value of work and have a job.

#### 4.3.4. Educator-Level Attainment Value

Educators also recognized other dimensions of attainment value in IBCs, describing the exams as “just another thing we have to check off the list,” as a “point” needed for graduation or a “check mark” for accountability purposes. Because of the funding tied to accountability ratings and CCMR indicators, in some cases, educators were even given a “list of students who [they] were responsible for certifying” by the end of the year from their school or district, making students’ performance on the exams of personal importance to the educators. This pressure on teachers can translate to pressure on students, with some high schoolers saying, “they definitely push it” and knowing that “the school wants everybody in CTE to graduate with at least one certification.”

#### 4.3.5. Student-Level Utility Value

Educators see immense utility value in their CTE courses, which provide students with hands-on learning experiences that mirror industry skills and experiences, “validate students’ pathways and purposes,” and train students for in-demand, high-paying jobs. They echoed students’ sentiments about the value of building students’ soft skills in CTE coursework so that they could be successful after high school. Generally speaking, CTE teachers implied that these courses were more valuable for students whose current and long-term career goals did not involve postsecondary education; however, they felt the soft skills and community that students built in CTE courses would serve all students’ personal and professional goals.

For particular certifications, where educators can clearly see the parallels between the exam and the profession, educators felt that IBCs “make [students] marketable right outside of high school.” For instance, one teacher noted that “for CMA (the Certified Medical Assistant exam), I think it’s great. I think they did a really good job of making sure that the curriculum is going to almost mimic the NHA (National Health Career Association exams).” In these cases, the CTE teachers feel the IBC is recognized as a valuable certification by the professional community or is directly parallel to a professional credential in that field. However, teachers note that this “depends on the CTE course,” and not all certifications guarantee that students will be able to “immediately start a relatively good paying job.” Some IBCs are described as “filling some space [on their resumes] at the very least,” indicating educators see minimal utility value in certain certifications.

While CTE departments often offer IBCs that are formally aligned to the coursework and industry sectors, there is a consensus amongst educators that IBCs are not always aligned to what is valued in industry or valuable to students. The CMA exam, for instance, was referred to as “the exception” rather than the norm. One teacher in Automotive Technologies noted feeling

“a sense of defeat. The IBC that they want me to teach has value, but it’s… it’s not going to get the student to stand out to [employers] more. It’s just as simple as that. I know that. I came from the industry. The IBC that I [teach] is geared to another level, more down the line of being an owner. It does have value… It has value, no doubt. But it’s not the value that’s going to stand out when the student hands in a resume [for an entry-level job], and it’s not going to make them stand out compared to a student that will walk in with the IBC I want to give them.”

Several other teachers felt this same tension while talking to their students about the value of the IBCs, saying that it’s “walking a fine line,” and “even though [they] don’t agree with the value [students are] getting, [they] still try to emphasize that it is a value to them,” they “sugarcoat it.”

#### 4.3.6. Educator-Level Utility Value

This sense of obligation to overstate their beliefs about the utility value IBCs hold for students may be due to external pressures that create a secondary form of utility value of IBCs for educators, where IBCs are a means for accomplishing school-level accountability goals and, at times, are metrics for incentive allotments or indicators of teacher performance. Several educators noted that “your numbers as a teacher, that is tracked within CTE. They are always like “You have to be doing better every year in terms of number of certifications.” Other educators received more specific directives from their districts about which courses and which IBCs to offer and promote, regardless of if they felt the courses and IBCs held utility value for their students:

“things like flower arrangements class. Not sleeping on that, it’s a great job, but I just don’t see how realistic that is for our class of students. But that was a big focal class on campus. … I think if we didn’t get any funding for it, I don’t think they would push it as hard. I don’t think we would be getting called with, “You need to have this many students in this class.”

These tensions led educators to pose questions like: “Are we doing it for the kids, or have we turned into a business about school?” Other educators remarked that the IBC exams are “not a graduation requirement, but it makes or breaks a district for a grade, and these are things that are not best practice for kids. We’ve lost sight of what is right for our kids.” While they’d prefer to prioritize what students need, one counselor remarked that “I work for our community. I also work for our district, and I try to… it’s hard to play both. It’s hard to play both.”

#### 4.3.7. Student-Level Costs

CTE teachers generally felt their courses provided students with low-cost, high-reward opportunities for skill attainment. However, educators expressed frustrations with their schools, districts, and the state, pointing out that accountability metrics and the cost of IBC exams appeared to dictate which IBCs were available in which CTE courses – rather than the value of the IBCs for students. In petitioning their district for specific IBCs, one teacher recalled that “they’ll literally ask you at the beginning of the year, ‘Submit the IBCs you want.’ You do, and they’ll come back with, ‘Hey, can we do this other one over here? This first one’s more expensive. This second one’s a little bit more in our budget.’”

Teachers similarly felt frustrated with the statewide entity, TEA, that creates the list of IBCs, acknowledging that regardless of a certification’s value to students, the school or district “won’t pay for it if it’s not on that list” and “our hands are tied with what we can choose.” Many teachers felt that there were extremely valuable certifications that were not included on the TEA list. In IBC selection, educators faced conflicts between school, district, and state policies, budget limitations, curriculum constraints, and the goal of offering students the most beneficial certifications, saying “we butt heads with what we think is going to provide the best value to the student educational-wise versus money and what they want in the curriculum.”

Educators also faced drawbacks in incorporating IBC exams and test preparation into their courses; increasingly, the CTE teachers were feeling that the IBC exams were forcing them “to teach to the test,” shifting class time away from hands-on learning and projects – the very components of CTE that students and teachers cite as the most valuable. This was especially frustrating to teachers who felt that:

“as far as how important [IBCs] are in the industry, that’s where it gets complicated because … I think it’s not really as important as the district makes it. Unfortunately, it also alters my curriculum in a way that I’m not real comfortable with.”

#### 4.3.8. Educator-Level Costs

In addition to feeling these curriculum modifications harmed students’ learning experiences, modifying course content and teaching test-taking skills also increased educators’ workloads. These modifications are in addition to the time and financial costs associated with coordinating curricular experiences and testing. For instance, programs requiring off-campus testing, such as Criminal Justice, placed additional burdens on teachers, where one instructor “had to load them up on a bus myself and take them [to get fingerprinted for the certification] … It’s not an easy process.” Teachers described taking on the additional responsibility of raising funds for student learning experiences that were not covered by their school or district, saying “they said that they were going to start compensating us partially for it, but there’s still transportation or, “Okay, well, the kids who are going to need uniforms. Anybody? Anybody? No. Okay. You’re going to have to fundraise.” In order to maximize student participation in IBCs, teachers felt they:

“have to make sure you’re coming to every single after-school event, parent-teacher night [to recruit students], which I do. I do that. But for the things that they’re wanting out of me, they are not being supportive of that. I’m sure I’m not the only teacher that’s saying that piece of it. It’s bad.”

## 5. Discussion

Why people are motivated to pursue an activity is one of the most enduring questions in social science. EVT, arguably the most prominent framework for studying motivation, elucidates that individuals’ ESs and EVTs are key components of their motivation. EVT scholars have examined how socializers influence the motivation of others and, to a lesser extent, how the “cultural milieu” enables and constrains socializers’ influence, resulting in a broader SEVT framework that situates socializers and those whom they motivate within this environment. However, limited SEVT research has engaged with how policy forces form part of this cultural milieu and, in particular, how examining them may help to address a seldom acknowledged mechanism: how socializers motivate others to complete a task, even if they do not perceive value in the activity.

In this study, we developed the concept of “situated sugarcoating” in order to describe this phenomenon. Much of the rationale behind the growing emphasis of IBCs in state and federal CCR policies relies on an assumption that these certifications offer clear and tangible utility value to students. While students saw the IBCs as beneficial for meeting their goals, educators described truly valuable certifications as “the exception” rather than the norm and expressed a “sense of defeat” over which IBCs were available to students. Despite these doubts and clearly identified costs of incorporating the exams into CTE programs, educators expressed a sense of obligation to “sugarcoat” the value of IBCs because of the salient accountability and funding environment. We next review the alignment between our findings and prior literature, describe the potential policy implications of this work, and finally, discuss the application of the theoretical concept of situated sugarcoating in other educational contexts.

### 5.1. Students’ Perceptions

Students consistently described the expectancy, intrinsic, attainment and utility values of their CTE coursework, and view IBCs as a welcome addition with expectancy, attainment and utility values. Our findings documenting gains in expectancies for success align with prior CTE literature that suggests that completing CTE courses and IBCs can boost students’ self-efficacy (Spillers & Lovett, 2022), and the broader literature base on self-efficacy, where mastery experiences like the hands-on learning experiences built into CTE coursework play a crucial role in building students’ efficacious beliefs (Bandura, 1997). Our findings are also consistent with CTE literature that showed students recognized intrinsic value in the practical, hands-on nature of the classroom experiences (Hayes, 2021) and highlighted the attainment and utility value students place on successfully completing CTE programs (Hayes, 2021; Newman, 2024).

Uniquely, this study identified that students also see attainment and utility value in successfully completing IBC exams, believing these credentials will give them a competitive advantage in the workforce. Notably, this perspective was shared even amongst students planning to pursue postsecondary education, who viewed IBCs as a valuable backup plan or a pathway to securing part-time jobs in their field that could help finance and supplement their studies. Regretfully, due to the lack of demographic information about our sample, we are unable to explore how perceptions of CTE and IBCs may vary across race, gender, or socioeconomic background; future research should explore this variation between students’ characteristics and across various state policy contexts.

Generally speaking, students’ perceptions of the values of these experiences suggest that both CTE and IBCs provide affective and motivational benefits linked to educational and career persistence, a perspective which has been largely absent from quantitative evaluations of these programs. However, these beliefs appear to be informed by sugarcoating from educators, situated in the broader CCR policy context which creates educator-level dimensions of value that supersede their beliefs about student-level value.

### 5.2. Educators’ Perceptions

Educators echoed many of students’ sentiments about the student-level value of CTE, but did not ascribe as much student-level value to IBCs. Because certifications with utility value towards students’ long-term goals are seen by educators to be few and far between, there is a general consensus that, on net, the addition of IBCs actually dampens the student-level values of CTE experiences. Perhaps in spite of these reservations, educators also identified educator-level attainment and utility values in IBCs, with some added costs, via their function in meeting certain CCMR benchmarks for accountability and teacher evaluations. These beliefs led some educators to “sugarcoat” the value of IBCs to students, in order to incentivize IBC participation and meet their own goals. Here, educators appear to be developing a hierarchy of the relative importance of student- and educator-level ESs and STVs that is “both situationally specific and culturally bound” in the broader CCR policy context. (Eccles & Wigfield, 2020, p. 2).

In doing so, beliefs about the educator-level values of IBCs are superseding their beliefs about the lack of student-level value, and leading to misrepresented or overinflated promotion of IBCs. This is concerning, given that students seem to heavily rely on the recommendations and expertise of their CTE teachers to determine the relative values of IBCs, as opposed to mentioning job postings or labor demand as motivation for IBC receipt. This mirrors patterns in extant literature, where educators can unintentionally misrepresent the value of certain college and career readiness options to students, based on their own biases (Cashdollar, 2023). The misrepresentations then function as a “push,” outside of students’ own goals and motivations, which encourages certification receipt via institutional pressures and policy levers (Dalton et al., 2021, p. 6).

### 5.3. CCR Policy Implications

Overall, our findings reveal a complex dynamic between students, educators, and policy in terms of the perceived value of CTE and IBCs. Incentivizing students’ receipt of IBCs risks prioritizing quantity over quality, and educators suggest that the incorporation of certifications into CTE courses detracts from the value of CTE learning experiences. Districts may evaluate the performance of CTE teachers based on their students’ completion of IBCs; for example, the Texas Incentive Allotment (TIA) allows districts to choose measures to evaluate teachers, and some districts have chosen IBC scores as a student outcome for this purpose. This could drive educators to select certifications that are easier for students to pass or less intensive to prepare students for (e.g. a CPR certification, rather than a nursing license), in order to boost their own evaluations.

At the school or district level, incentives may lead to gaming accountability systems. A school may react to CCR policies by incorporating easy to earn certifications (e.g. a Floral Design certification) into required courses, thereby certifying the vast majority of its students with something that likely provides little professional value. If schools are gaming accountability plans and funding systems by overprescribing irrelevant IBCs to students, then students waste time and energy preparing for and taking these exams, teachers waste preparation and classroom time teaching IBC content or test-taking skills, and school, district, state, and federal resources are wasted in the processes of selecting, equipping, funding, and administering exams that provide no value to students. These policy responses may vary by school-level characteristics that we were unable to capture in this study, such as the demographics of its student population or recent accountability ratings. For instance, resource-constrained schools may have more motivation to push certifications and maximize funding, or low-achieving schools or schools that have struggled to meet accountability requirements may be more likely to push certifications for CCR points. Future research should investigate variation in the prevalence of situated sugarcoating across school and student characteristics.

Educators shared frustration that state or district policy priorities push them towards selecting cheaper certifications; policymakers could consider restructuring IBC funding so that the direct cost to the school or district is more equal across certifications. Alternately, it could be beneficial to assign each certification a utility value ranking, and subsidize costs to be equal within levels (i.e. level one certifications cost $50 per student), or scale the proportion of the certification costs that are covered by level (i.e. level one certifications cost $50 and level 3 cost $150 per student). Some states have implemented policies that provide more accountability “points” for IBCs that require more education and training than others. For example, earning a license as a licensed vocational nurse requires far more coursework than completing a CPR certification. Although both would count as health science IBCs, it is reasonable to offer stronger incentives for schools to support students’ completion of higher-level IBCs. Future research should investigate ways to assess the value of specific credentials, relying on both the perspectives of students and educators, as well as quantitative models determining the relationship between specific IBCs and students’ educational and workforce outcomes.

### 5.4. Theoretical Contributions

We propose that the situated sugarcoating of IBCs documented here is not specific to only this sociopolitical context, but may be a key mechanism that links educational policies with student motivation across various contexts. Although our empirical case is grounded in the CCR policy context, the underlying process has broader theoretical utility. SEVT posits that socializers’ beliefs and values shape the motivational beliefs of the socialized, but generally conceptualizes the socializers’ beliefs as authentic. Situated sugarcoating builds upon this theory by foregrounding how sociopolitical contexts can disentangle socializers’ values from the values they publicly communicate.

Situated sugarcoating has four components: (1) socializers (e.g., educators) do not personally see an activity as valuable for those they guide; (2) broader social, economic, and political contexts frame the activity as valuable, particularly for the socializers; (3) these pressures lead socializers to encourage participation despite their own doubts; and (4) recipients value the activity and are motivated to pursue it. We argue this phenomenon is distinct from other theories that interrogate the relationship between policies, motivation and action. For instance, while the concept of controlled motivation within self-determination theory also implicates the role of socializers in shaping student motivation (Deci & Ryan, 2012), situated sugarcoating extends this idea by exploring how sociopolitical contexts influence the behaviors that socializers seek to motivate students to accomplish, even when socializers do not see inherent value in those behaviors or attainments. And unlike (de)coupling or policy feedback theories (e.g., Coburn et al., 2016), which connect political pressures and institutional or educator responses sugarcoating incorporates the experiences of the students influenced by these mechanisms. Importantly, situated sugarcoating implies dissimulation, where socializers do not reveal their true motives for encouraging others’ behavior, which prior motivational theories have rarely addressed.

Situated sugarcoating likely occurs in other educational and non-educational settings, when those with potential influence over others are embedded in social environments that create incentives for them to motivate behaviors that they do not value, either in general or for specific individuals whose behavior they are guiding. Potential examples include school counselors motivating students to pursue higher education due to school accountability pressures even if they believe a student’s chances of succeeding in college are low; professors encouraging students to explore “diverse perspectives” of settled scientific issues (e.g., human evolution) due to policy pressures; medical providers encouraging patients to consider additional tests or procedures that have limited medical value due to insurance incentives; and managers motivating employees to complete tasks that bolster the manager’s performance metrics, even if they provide no benefit to the employee. While potential examples in educational contexts abound, future research should explore the other contexts in which situated sugarcoating occurs and why.

### 5.5. Limitations

While this study offers valuable insights into how students and educators perceive the value of CTE and IBCs several limitations should be acknowledged. First, we did not collect demographic information from participants. These factors may significantly shape students’ career aspirations, access to resources, and perceived value of educational opportunities. As such, we are unable to assess whether and how perceptions of CTE and IBCs differ across lines of identity or background. Second, although our sample included students, educators, and administrators across seven Texas high schools, the relatively small number of participating schools limits the generalizability of our findings. A broader sampling of schools could enable more robust comparisons and a deeper understanding of how institutional and community characteristics shape perceptions of value. Similarly, our educator sample was not large enough to support fine-grained comparisons across different staff positions. In particular, perspectives may vary meaningfully among CTE teachers, school counselors, CCMR specialists, and administrators. Future research could benefit from a larger and more role-stratified educator sample to better capture intra-school variation in beliefs and practices related to CTE and IBCs.

## 6. Conclusions

Amid the increasing prioritization of IBCs in state and federal CCR policies, we sought to investigate how students describe their motivation for participating in CTE and IBCs, how educators interpret the value of these experiences and in turn, shape students’ beliefs, and how the broader CCR sociopolitical context may shape these dynamics. We find evidence that educators engage in situated sugarcoating, where they overrepresent the value of certain educational opportunities, like earning an IBC, due to the political pressures which make the student engaging in the activity valuable to the educator themselves. This sugarcoating occurs despite educators questioning the student-level value of the IBC, but ultimately results students both participating and perceiving value in their experiences. It remains uncertain whether students’ perceived value reflects an exaggeration of limited benefits, the absence of meaningful value altogether, or whether the affective reassurance produced by sugarcoating confers advantages (i.e., a placebo effect) that may outweigh its costs. However, understanding the perspectives of those closest to the classroom—students and educators—offers a critical step toward more equitable and effective CCR policy. Beyond its applications in CRR policy, the concept of “situated sugarcoating,” where educators over-emphasize the benefits and understate the costs of educational opportunities due to the particular social, economic, and policy environment, is a theoretical contribution of this work. We encourage other authors to engage with the politically situated nature of value and motivation in education settings, particularly given the large quantity of policy-related research that relies on quantitative rather than qualitive work.

## Figures and Tables

**Table 1:** Research Participants and School Characteristics

	Site 1	Site 2	Site 3	Site 4	Site 5	Site 6	Site 7
**Participants**							
Students	9	8	12	16	6	12	5
Educators	2	7	9	7	3	6	10
Administrators	1	0	3	2	2	4	2
**School Characteristics**							
Campus Size	226	1,825	1,258	2,154	469	99	2,089
Region	City	City	Rural	City	City	City	Rural
IBC Completers	69%	2%	20%	43%	49%	73%	53%
CTE Participators	86%	91%	100%	99%	98%	100%	100%
CTE Concentrators	9%	17%	61%	64%	4%	100%	51%
Advanced Course Participators	59%	80%	47%	72%	100%	29%	68%
DC Participators	18%	45%	27%	20%	98%	92%	7%
**School Demographics**							
Female	56%	48%	45%	44%	62%	25%	50%
Asian	≤2%	≤2%	≤2%	≤2%	≤2%	≤2%	≤2%
Black	6%	7%	10%	≤2%	≤2%	10%	20%
Hispanic	56%	67%	29%	95%	93%	71%	60%
Native American	3%	4%	2%	≤2%	≤2%	≤2%	≤2%
Native Hawaiian or Pacific Islander	≤2%	≤2%	≤2%	≤2%	≤2%	≤2%	≤2%
Multiracial	≤2%	≤2%	≤2%	≤2%	≤2%	≤2%	≤2%
White	34%	21%	57%	3%	7%	16%	19%
Economically Disadvantaged	71%	63%	45%	63%	50%	100%	53%

**Table 2:** Summary of Findings across SEVT Components

	Students		Educators		
	CTE	IBCs		CTE	IBCs
***Expectancies for Success***	High	Medium		High	Medium
*Student Level*	Confidence in soft and technical skills; Confidence from community and mentorship; Confidence in their ability to succeed in their field	Confidence in their ability to get a job		Confidence in soft and technical skills; Confidence from community and mentorship; Validating students’ post-graduation plans	Students’ confidence in their ability to get a job
*Educator Level*	-	-		-	-
*Example Quote*	“I would confidently say, if I would have to go be a cop right now, I could be confident in riding along with someone.”	“You know what you’re doing, you got an idea. And that can put you ahead of somebody else.”		“I think programs like ours validate what they really want to do, what their pathway and purpose is.”	“Some of the kids get a little bit of a boost out of it, an ego boost.”
***Intrinsic Value***	High	Mixed		High	Low
*Student Level*	Hands-on and interactive projects; More enjoyable than other courses; Sense of community with peers with shared interests	Interesting when exams are hands-on and creative applications of skills; --- Addition of test preparation dampens enjoyment		Hands-on and interactive projects; More enjoyable than other courses; Students’ sense of community with peers with shared interests	Limits or removes hands-on and interactive projects from curriculum
*Educator Level*	-	-		-	-
*Example Quote*	“it doesn’t feel like a class. Class is boring. I would compare it to recess”	“test prep” and “book work”		“the creativity, the ability to be relaxed and let them actually apply [their skills]”	“the application is missing because I’m trying to teach to this test”
***Attainment Value***	High	High		High	High
*Student Level*	Importance of gaining skills relevant to their career interests	Importance of gaining a license required within their field		Importance of gaining skills relevant to their career interests	Importance of students gaining a license required in their field; Students receiving recognition for their achievements
*Educator Level*	-	-		-	Importance of passing IBCs for accountability and funding purposes
*Example Quote*	I think [we’re] motivated by that chance to make money off of whatever [we’re] doing, especially when [we’re] doing what they love.”	so I can “open my own shop after high school”		“we’re realists, and that’s what we try and give the students - real world experiences.”	“just another thing we have to check off the list”
***Utility Value***	High	High		High	Mixed
*Student Level*	Career exploration; Skill development within chosen career fields; Soft and professional skills	Skill development within chosen career fields; Skill signaling on their resumes; Back-up plans		Career exploration; Skill development within chosen career fields; Soft and professional skills	Some IBCs signal marketable skills; --- Others are not valued in industry
*Educator Level*	-	-			Educators must hit certain IBC or CCMR benchmarks for accountability and teacher evaluations
*Example Quote*	“a class that I know that I’m going to use after this that will lead me to where I want to be. And so, it’s a little more rewarding.”	a “leg up in the real world”		“They can go and start working in that profession, whether it’s a security officer, or pharmacy technician, whatever pathway they followed.”	“it’s not the value that’s going to stand out when the student hands in a resume, and it’s not going to make them stand out compared to a student that will walk in with the IBC I want to give them.”
***Cost***	Medium	Mixed		Mixed	High
*Student Level*	Curricular tradeoffs; Time spent learning and mastering course materials and skills	Time spent learning and mastering exam skills; --- Uncertain financial burdens; Sunk time investment lost via policy changes		Students can gain valuable skills at low-cost	Removes hands-on and interactive projects from curriculum
*Educator Level*	-	-		Costs not covered by the schools can fall to teachers to fundraise or supplement	Financial burdens; Extra time spent working outside of school hours; Risk of losing licenses or certifications; Frustrations with policy decisions on which IBCs can be offered
*Example Quote*	“you can’t really switch into something else”	the policy change “discounted all of the work that my friend and I had done”		“Okay, well, the kids who are going to need uniforms. Anybody? No. Okay. You’re going to have to fundraise.”	“we butt heads with what we think is going to provide the best value to the student educational-wise versus money”

## Educational impact and implications statement:

This article extends the lens of Situated Expectancy-Value Theory (SEVT) to document policy influences on educator and student behavior and motivation. We find evidence that educators engage in Situated Sugarcoating, where they overrepresent the value of certain educational opportunities, like earning an Industry-Based Certification (IBC), due to the political pressures which make the student engaging in the activity valuable to the educator themselves. Following the advice of their teachers, students see value in and participate in these experiences, and educators benefit.

## Appendix

**Table A1:** Student Focus Group Protocol & Theoretical Alignment to SEVT

#	Question	Theoretical Concept(s)
1	To get started, can we just go around and could you tell us your name, your grade level, what CTE courses you’ve taken, and then what you’re planning to do after you graduate high school?	N/a
2	Can you tell us about how you chose which courses you took this year? Did you talk to anyone else about which courses to take? Were you thinking about what you want to do after HS while choosing courses?	Expectancies for Success, Intrinsic Value, Utility Value, Attainment Value, Cost
3	What about the CTE classes appealed to you before taking the course? Was the subject of this course something you’re interested in doing after HS? What did you think you would get out of taking (course title)? Were you considering taking any others? If yes, how did you choose this course over other options? Are any of you working towards completing a CTE pathway?	Expectancies for Success, Intrinsic Value, Utility Value, Attainment Value, Cost
4	Can you tell me about your experience in your CTE course(s)? What was a typical class period like? What kinds of things did you learn or work on in your CTE course(s)? Were you able to choose your own projects?	Expectancies for Success, Intrinsic Value, Utility Value, Attainment Value, Cost
5	What were your experiences with your classmates and teacher like? How did you typically interact with your teacher and classmates? Did this classroom feel different than other classrooms? If so, why? Did you feel that the course met your individual needs and goals? Did you feel that the course met your peers’ needs and goals?	Expectancies for Success, Intrinsic Value, Utility Value, Attainment Value, Cost
6	Did you see value in the types of things you did or learned in (course title)? Did you find the coursework interesting? Did you enjoy being in (course title)? Did you feel confident in your ability to complete the coursework? Was it important to you to do well in (course title)? Do you think the things you learned in (course title) will help you after HS?	Expectancies for Succes, Intrinsic Value, Utility Value, Attainment Value, Cost
7	Did you take any certification exams as a part of your CTE coursework? How did you first hear about certifications? How were the certifications described to you? Based on your experiences and what you’ve heard about certifications and credentials, how would you describe those opportunities?	N/a
9	Were you able to decide with exams you took? Were you required to take the certification exam as a part of your CTE class? Did you take any certification exams outside of your CTE course subjects? Were you working towards a series of certifications or a variety?	Expectancies for Success, Intrinsic Value, Utility Value, Attainment Value, Cost
10	How would you describe the value of taking the certification exam? Did you find the certification interesting? Did you enjoy taking the (Certification title) exam? Did you feel confident in your ability to complete the exam? Was it important to you to do well on the exam? Do you think the certification will help you after HS?	Expectancies for Succes, Intrinsic Value, Utility Value, Attainment Value, Cost
11	What do you plan to do after you graduate from high school? Did completing this course change any of your plans for after HS? Did completing a certification change any of your plans for after HS?	Expectancies for Succes, Intrinsic Value, Utility Value, Attainment Value, Cost
12	Do you feel prepared for your post-graduation plans? Did either the course or certification change that for you?	Expectancies for Succes, Attainment Value, Utility Value, Cost

**Table A2:** Educator Focus Group Protocol & Theoretical Alignment to SEVT

#	Question	Theoretical Concept(s)
1	Can everyone start by telling us your name, CTE courses and programs you teach, how and long you’ve taught those courses here?	N/a
2	What was your pathway to becoming a CTE teacher? How long have you been teaching? Teaching CTE specifically? Did you work in an industry related to the CTE courses you teach prior to becoming a high school teacher?	N/a
3	How has the emphasis on CTE coursework or their prominence at this school changed over the years? Has CTE at this school changed much over the years? Have you noticed any changes in which courses are offered over the years? Or any changes in student enrollment in courses over the years?	N/a
4	How would you describe the process of how entering ninth graders choose the courses they’ll take in high school? Do they speak to a counselor about course selection? Are they required to take any CTE courses or pathways?	Expectancies for Success, Intrinsic Value, Utility Value, Attainment Value, Cost
5	Do teachers recruit students into courses and programs? What strategies do you personally use to recruit students into your courses and programs? What qualities in your students do you look for when deciding whom to recruit?	N/a
6	Why do you think students decide to take CTE courses? Do you think they find CTE coursework more interesting than other courses? Do you think students enjoy CTE courses more than their academic courses? Do you think they see more value in CTE coursework than academic courses? What is most valuable for students about their CTE coursework?	Expectancies for Success, Intrinsic Value, Utility Value, Attainment Value, Cost
7	How would you describe the purpose of CTE?	Expectancies for Success, Intrinsic Value, Utility Value, Attainment Value, Cost
8	In the CTE courses you teach, how are the goals and learning objectives of the courses determined? How do the TEKS factor into course learning objectives? How do the IBC exams factor into course learning objectives? Are students able to set their own learning goals in these courses? Do you incorporate goals and learning objectives from any other sources? How do you adjust your teaching to meet the different learning needs of students in your courses?	Expectancies for Succes, Intrinsic Value, Utility Value, Attainment Value, Cost
9	How do you decide what curricular materials you use in your CTE courses? Does your school or district influence which curricular materials you use? Do the IBC exams influence the curricular materials you use or teach? How would you assess the quality of the curricular materials you use? How much have you designed the curriculum yourself?	Expectancies for Succes, Intrinsic Value, Utility Value, Attainment Value, Cost
10	How much professional development have you received for the CTE courses you teach? Who decides what professional development you receive? How would you describe the quality of the professional development? Have you received any training or support on how to differentiate your course to accommodate students with diverse learning needs?	Expectancies for Succes, Intrinsic Value, Utility Value, Attainment Value, Cost
11	How would you describe the culture of your CTE classrooms? What was a typical class period like? How would you describe students’ relationships with each other in these courses? Do you see any differences between your CTE classrooms and non-CTE classrooms? If so, how?	Expectancies for Succes, Intrinsic Value, Utility Value, Attainment Value, Cost
12	What do you think students get out of their experience in CTE? Do you think the things they learn in your course will help them after HS?	Expectancies for Succes, Intrinsic Value, Utility Value, Attainment Value, Cost
13	How would you describe the purpose of the certification exams? What do you think students get out of taking a certification exam? How does that compare to other CCMR options like AP or dual-credit courses? Have your perceptions of the value of certifications changed over time? Why or Why not?	Expectancies for Succes, Intrinsic Value, Utility Value, Attainment Value, Cost
14	How do you think the certification exams are valued in industry? Are certifications prominent in your industry sector? Are there other certifications or credentials that are more valued in industry?	Utility Value, Cost
15	How do you think administrators in your school and district value certifications? Do you think they are valued or a priority for administrators? How does that compare to other options like AP courses, dual-credit courses, etc.?	Utility Value, Attainment Value, Cost
16	Is there anything else about CTE or the certifications that you’d like to add?	N/a

**Table A3:** Counselor & Administrator Focus Group Protocol & Theoretical Alignment to SEVT

#	Question	Theoretical Concept(s)
1	Can everyone start by telling us your name, your role, and how long you’ve been in that role at this school?	N/a
2	Can you tell us about what kinds of CCMR opportunities are available to students here?	N/a
3	How are decisions about which CCMR opportunities are available to students made at this school? To what extent does your district influence these decisions? To what extent do state entities (e.g. TEA) influence these decisions? To what extent do student preferences influence these decisions?	Intrinsic Value
4	Have you faced any barriers in trying to implement a particular CCMR programs? Is there any support your district could offer to better assist you? Is there any support state entities could offer to better assist you?	N/a
5	How has the emphasis on CCMR opportunities or their prominence at this school changed over the years? Have you noticed any changes in which courses or programs are offered? Or any changes in student enrollment in courses over the years?	Expectancies for Success, Intrinsic Value, Utility Value, Attainment Value, Cost
6	How would you describe the process of how entering ninth graders choose the courses or programs they’ll take in high school? Do they speak to a counselor about course selection? Are they required to take any specific CCMR offerings? Do teachers recruit students into courses and programs? How do you chose which CCMR opportunities to recommend to different students?	Expectancies for Success, Intrinsic Value, Utility Value, Attainment Value, Cost
7	How would you describe the purpose of: Career and Technical Education? Industry-Based Certifications? Dual Credit/Dual Enrollment courses? Advanced Placement/IB courses?	Expectancies for Success, Intrinsic Value, Utility Value, Attainment Value, Cost
8	What do you think students get out of their experience with: Career and Technical Education? Industry-Based Certifications? Dual Credit/Dual Enrollment courses? Advanced Placement/IB courses?	Expectancies for Success, Intrinsic Value, Utility Value, Attainment Value, Cost
9	Have your perceptions of the value of any of the CCMR opportunities changed over time?	Expectancies for Success, Intrinsic Value, Utility Value, Attainment Value, Cost
10	Is there anything else about CCMR that you’d like to add?	N/a
